# “Who will stand up for us?” the social determinants of health of women tea plantation workers in India

**DOI:** 10.1186/s12939-020-1147-3

**Published:** 2020-02-28

**Authors:** Preety R. Rajbangshi, Devaki Nambiar

**Affiliations:** 1 The George Institute for Global Health, 311-312, Third Floor, Elegance Tower, Plot No. 8, Jasola District Centre, New Delhi, 110025 India; 2 Public Health Foundation of India, Institutional Area, Plot 47, Sector 44, Gurugram, New Delhi, 122002 India

**Keywords:** Women workers, Health, Tea plantations, India, Social determinants of health

## Abstract

**Introduction:**

The tea estate sector of India is one of the oldest and largest formal private employers. Workers are dependent on plantation estates for a range of basic services under the 1951 Plantation Labour Act and have been subject to human rights violations. Ad hoc reports related to poor health outcomes exist, yet their determinants have not been systematically studied. This study in Assam, situated in Northeast India, sought to understand the Social Determinants of Health (SDH) of women plantation workers with an aim to offer directions for policy action.

**Methods:**

As part of a larger qualitative study, 16 FGDs were carried out with women workers in three plantations of Jorhat district covering permanent and non-permanent workers. Informed consent procedures were carried out with all participants individually. Data were analyzed thematically using Ritchie and Spencer’s framework based on an adapted conceptual framework drawing from existing global conceptual models and frameworks related to the SDH.

**Results:**

Determinants at structural, intermediary and individual levels were associated with health. Poverty and poor labour conditions, compounded by the low social position of women in their communities, precluded their ability to improve their economic situation. The poor quality of housing and sanitation, inadequate food and rations, all hampered daily living. Health services were found wanting and social networks were strained even as women were a critical support to each other. These factors impinged on use of health services, diet and nutrition as well as psychosocial stress at the individual level.

**Conclusion:**

Years of subjugation of workers have led to their deep distrust in the system of which they are part. Acting on SDH will take time, deeper understanding of their relative and/or synergistic contribution, and require the building of stakeholdership. Notwithstanding this, to have heard from women workers themselves has been an important step in visibilizing and building accountability for action on the health and SDH of women in plantation estates.

## Background

India’s tea industry is one of the oldest private employers in the world. In the North Eastern state of Assam, nearly one in five persons is employed in the plantation sector [1], relying on estate for employment as well as a range of services including housing, water, health, education, and many facilities that affect the daily lives of worker [2]. This has been historically shaped by the practice, by British companies since the nineteenth century, of coercively bringing low caste (Dalits or untouchable caste), poverty-stricken, landless and tribal (Adivasi or Indigenous) populations from central and southern India to the state, since local agriculturalists refused to work on plantations. Women were key to this process, ensuring social reproduction and regeneration of the workforce [3], in addition to performing select tasks (like plucking) with greater quality [4]. Once India gained independence, in 1951 a Plantation Labour Act (PLA) legitimise a key – and exploitative [5] - occupational feature of plantations in the colonial era: wages have remained low, because in-kind benefits related to housing and social welfare are to be provided to workers as per the Act [6]. The net effect has been stasis: as in the colonial period, tea plantations continue to control the lives of their workers in a parallel governance structure, with little active involvement by the State [6]. Workers over generations remain landless and landlocked, facing myriad forms of marginalisation.

While poor conditions of plantation workers and non-compliance of PLA in Assam have been documented by several national and international organizations [7–9], far less is known about the health and well-being of women workers. What is known is that more than 50% of the labour force in plantation estates is women as it is believed that “soft hands and nimble fingers of women” are suited for plucking tea leaves [10]. Studies also suggest that women in plantation estates are more likely to have severe anaemia, be married early and have high parity [11–14].

In 2009, knowledge of the high burden of maternal deaths (237 per 100,000 live births in Assam as against 130 per 100,000 live births national) [15] in the state motivated the health department to intervene and provide health services in plantation estates. Nevertheless, there remains a broader lack of understanding of women worker’s conditions and their lives and rights – their socioeconomic position, conditions of work, access to health and education - the Social Determinants of Health (SDH). Our study sought to address these gaps by understanding women workers’ conditions, their needs, and perceptions related to their health and well-being – from their perspective.

## Methodology

A qualitative descriptive study was conducted in the state of Assam. Field-based data collection was conducted in the fall of 2016. Formal permissions were obtained from the district administration where this study was undertaken, as well as the management of each of the plantation estates from which workers were recruited [names have been withheld to protect plantation estate’s anonymity as per ethical requirements and obligations]. The selection of tea plantation estates was purposive and based on consultation with district health officials: one government tea plantation and two private sector plantation estates were chosen.

The participant selection strategy for focus group discussions (FGDs) was developed by a team of researchers and involved criterion sampling designed to reflect particular working conditions and entitlements. For one, permanent and non-permanent women workers were studied separately. Sub-groups were selected based on years of service in tea plantations. Based on this, the following groups were formed (a) women who had worked in the tea plantations for less than a year; (b) women who had worked in the tea plantation for 1–5 years; and (c) women who had worked in the tea plantation for over 5 years. Focus groups sought to understand shared perspectives on health and its determinants, women workers’ needs as well as experiences with existing services.

### Data collection and analysis

Women were contacted and explained the purpose and ethical aspects of the study. We also informed them that approvals from management were taken and that there was no threat to their daily entitlements, job security, and retention. On acceptance to participate, groups were formed based on our selection criteria. Permission to record the discussions was taken from each participant. A total of 16 FGDs were conducted.

Soon after each FGD, field notes were written up by the researcher and digital recordings of discussions were stored in a folder protected from unauthorized access. Each recorded group discussion and interview was transcribed by a professional transcriber to obtain verbatim transcripts. Each transcription was overseen by the lead author and a quality check was performed. Transcripts were cleaned and entered into Atlas. Ti software. Based on Ritchie and Spencer’s Framework Method [16], we created a priori codes according to structural, intermediary and individual factors described in relevant frameworks on social determinants of health that we identified in advance of the study that were relevant to women’s health [17, 18]. Emerging codes were developed and compiled into the framework. Quotations were indexed and charted according to sets of codes. Codes and quotations were interpreted and associations were drawn in narrative and presented under themes corresponding to the levels – structural, intermediary, and individual.

## Results

### Participant profile

16 FGDs with women workers -- nine with permanent and seven with non-permanent workers were carried out. A total of 134 women took part in these discussions and ranged in the age group of 18 to 55 years. Women in the discussions were pre-dominantly married with small proportion either unmarried or widow. Table 1 summarizes the characteristics of participants.

**Table 1:** Distribution of FGD participants by age and contract type

Type of contract	Number of FGDs	Number of participants	Years of work experience	Average age (in years)
Permanent	3	22	Less than 1 year	29.5
	3	21	1–5 years	28.7
	3	27	More than 5 year	38.4
Non- Permanent	2	15	Less than 1 year	23.1
	3	33	1–5 years	25.8
	2	16	More than 5 year	31.3
TOTAL	**16**	**134**		

In all plantations, women informed us that their primary job was plucking tea leaves but they were also involved in manual jobs such as pruning bushes during winter, sprinkling what they called “dry white powder” in tea bushes (probably urea/pesticide).

Permanent workers were engaged by plantations around the year and were entitled to employee benefits such as housing, health services, provident fund, and gratuity. Non-permanent workers were engaged whenever required for plucking tea leaves. Typically, non-permanent workers were related to workers from the plantations (i.e. a wife, daughter or daughter-in-law) or from the villages surrounding plantations.

### How women plantation workers view their SDH?

There was a strong feeling among women that no one was genuinely interested in uplifting their community- whether in relation to health or its determinants. We found that women did not expect a lot from plantation management but felt entitled to some support from the state government. These expectations notwithstanding, the majority of the plantation populations were left out from government programs and schemes like this. We sought to link this larger perspective of women plantation workers – to known frameworks that categorize structural, intermediate and individual determinants of women’s health.

#### Structural determinants

### Income and poverty

All women across plantations noted that poverty was affecting a wide range of factors in their lives, including health. They noted that poverty in plantations was because of poor wages. While workers in private plantations reported receiving INR 126 per day (USD 1.8 per day), their counterparts in a government plantation were receiving a daily wage of INR 115 per day (USD 1.7 per day). In both cases, workers reported that the amount of money they earned was inadequate to cover household expenses.

All women noted that their wages were linked to daily work quotas of plucking 24 kg (kg) leaves. If they failed to meet this target, there would be wage deductions-ranging from INR 1 per kg (USD 0.014 per kg) to INR 5 per kg (USD 0.070 per kg).

With regard to social security, permanent workers were entitled to three months’ maternity leave after delivery whereas non-permanent workers had to leave employment altogether. However, during maternity leave, permanent workers were not given full payment and half of their daily wage was deducted. There was limited discussion of social security in general, although it was made known that permanent workers had provident funds and other benefits, while non-permanent workers did not.

### Literacy and education of children

Women in the groups were either illiterate or school dropouts. They explained that in the larger context of poverty, there prevailed a perception that girls should not be educated. However, all women acknowledged the importance of education and expressed eagerness in supporting their children’s education. While primary school was subsidized, women reported that the lack of support for education beyond this level meant that most children in plantations were not completing school. Instead, they would enter the workforce to supplement their parents’ earnings. Longer term, this would lead to similar vulnerabilities for their future generations.

### Social position in the community, at work and home

Women reported a bias in roles and position of men versus women in their community. The collective view was that women’s opinions did not matter at the community level. While men were free to shape and chose their roles, women’s roles were confined to their plantation labour and labour in the private domain, i.e. housework, child and elderly care.

Even at work, women reported neglect at the hands of management. As noted by a permanent worker “We are not valued. We are small people and they [management and supervisors] are big people, and they will only speak with big people. We would have discussed our problems [with them] if sometimes they [would] speak with us”.

Women also mentioned that they feared speaking out about or ask anything as they were the labourers and not staff. For instance, the precise logic or quantum of wage deduction (for not achieving a daily plucking quota) was not known by women. As one non-permanent worker mentioned: “They [management and supervisors] do not tell us how much is deducted. We will know only if they tell us. We fear to ask because they will scold us”. Thus, poverty was linked to a clear asymmetry in the knowledge and social position of women workers relative to others in their family, community, and work, across plantation types.

This missing voice of women was noted with respect to worker unions as well. Women from government plantations did not report a membership of or leadership in worker unions. Meanwhile, workers in a private plantation reported that their new union was formed with 17 men and four women members. When asked whether women members could become Union President, women were unfamiliar with the rules around this and expressed scepticism around the possibility that a woman could hold this post.

Some women reported that even as they were equal contributors to household earnings, they could not voice their opinions – at home nor in public discourses. Concurrently, a few women noted that they did participate in household decision-making but they are not the decision-maker. Most women concurred that they could not do anything without their husbands’ knowledge or against their will. Even decisions like whether to work or not or where to work were reportedly decided by their husband.

### Working conditions: supplemental and unpaid labour

Women across FGDs reported a range of paid and unpaid labour. In fact, women were engaged in labour at all times except when they were sleeping. Mondays through Saturdays were for plantation work. On Sundays, they supplemented their incomes through informal sector labour like brick kiln work, daily paid agricultural work or domestic work in nearby villages. Women (mostly permanent workers) who did not have paid work on Sundays, carried out household-related unpaid labour, such as collection of firewood for the week.

All women carried out a range of tasks as part of their domestic labour. For most, labour began in the morning 4 am and ended at around 10 pm, when they went to sleep. On working days, from 4 am to 7.30 am they would complete household chores and then report to work at 8 am. At work, they would get an hour lunch break from 1 pm to 2 pm, which they sometimes skipped to meet daily plucking targets, continuing to work until 4 pm. After reaching home, they would prepare dinner, fetch water and feed their families. After dinner and cleaning the kitchen, they would go to bed between 9 pm and 10 pm. This routine was followed by women irrespective of the type of plantation and contract.

A few women mentioned in jest that it was at the end of the day after lying on the bed to sleep that they feel the pain in their bodies. They noted that they were habituated to continuously working and therefore cannot sit idly at home.

### Working conditions: occupational health & safety

Women’s work continued through year, across seasons; for instance, tea plucking did not stop in times of rain or monsoon. Such work was especially difficult during the monsoon season because of the lack of a shelter for lunch break, risks of injuries due to slippery roads, and the effect rains would have on plucking quotas. For instance, a woman at a private plantation narrated an incident where she fell on the road and injured her wrist while carrying plucked leaves on her head. She was on her way to deposit them. She could no longer bend her wrist.

In another instance, a women’s group reported the death of five of their colleagues while plucking leaves during a thunderstorm. As there was no provision for them to stop working while raining, women informed us that they continue plucking leaves by covering their head with *Japi* (a traditional conical hat) or a plastic bag. There was pervasivefear of losing a day’s wage if the target was not met.

*“Leaves are wet and when we take the leaves for weighing… say if the leaves weigh 5 kg then they [collectors] will consider 3 kg, when we take 10 kg to weigh then they will consider 7 kg. Like this. They think because of water it is heavy, that’s why they reduce the weight [and accordingly calculate the daily wage]”*.

*Non-Permanent workers at a private tea plantation.*

On an ongoing basis, women reported susceptibility to occupational hazards such as insect bites. We came across an accident, in one plantation, where a woman worker was found unconscious after being bitten by bees. We also observed that for protecting themselves from insect bites, women across plantations carried a locally prepared emollient made of mustard oil, lime and tobacco leaves, which they repeatedly applied on their legs. Such strategies were adopted given the lack of protective gear such as gloves and boots.

*“We do not wear gloves. They [management] don’t give us. We sprinkle [urea/pesticides] with hands. Hands get swollen and fingers burn. If a thorn pricks, then it burns. You have seen, how thorny it is?”*

*Non- permanent workers at a government tea plantation.*

While discussing their working conditions, women stated that there was no provision of latrines or handwashing facility for them, and whenever needed, they would defecate in the plantation areas. Also, there was no provision for changing sanitary pads/cloths during menstruation at work. Women described changing pad/cloth under tea bushes, wrapping the used pad/cloth in a polythene bag and keeping it with them. This used cloth was then washed and kept for reuse. At a private plantation, women would go home to change their menstrual absorbent cloths during lunch break.

Concurrently, a few women also noted the need for a clean tank for distributing black tea and drinking water to workers working in the field. A woman in a private plantation mentioned that “Once we found a snail in the water, and since then we stopped drinking water [distributed in plantation area for workers] and started bringing [it] from home. But sometimes [when that water finishes] we drink this water [from the tank]”.

### Working conditions: crèche services

While discussing their working conditions, women workers also expressed the need for better crèche services. While day-care services were supposed to be available in each plantation estate, only one private plantation had fully functional crèche, for which only permanent workers’ children were eligible. In two other plantation estates, the management had designated a bamboo hut (without wall) as crèche with no facilities.

### Living conditions: housing and area of residence

Permanent workers were entitled to housing facilities but a few non-permanent workers were also residing in the labour lines because their husband or a family member was a permanent worker. Labour lines are the areas within the tea plantation estates premises designated for worker’s residence.

Women across plantations mentioned housing shortfall. A permanent worker stated “where we will go, this is our place. Our family lived in the plantation for generations”. A similar feeling was shared by a few non-permanent workers who were forced to leave the labour line and now they were staying in villages around the plantation.

A few women in government plantation mentioned not having electricity and water connection in their quarters. Almost all women across plantations mentioned that their complaints about repairing and renovation went unanswered, even as the buildings were decades old. While discussing housing, women noted that all permanent workers were not allowed quarters.

*“If two brothers are permanent, only one brother will get a quarter and the other [brother] must build his own house and stay. And if one of the brothers falls sick and needs to cancel his name [i.e. resign from the job], then he cannot work. Then, for his wife plantation should do something but they do nothing”.*

*Permanent workers at a private tea plantation.*

Along with quarter shortage, women mentioned inadequate latrines in labour lines. Therefore, most people in labour lines would defecate in plantation areas. A few women mentioned that it was difficult for them and children to go out at night and therefore sometimes they defecate in their compound and clean the waste in the morning.

Also, women admitted the inconvenience to pluck leaves, particularly during monsoon season, because of the pervasive smell of human excreta. Those who had latrine at home and staying at labour lines mentioned that latrine tanks were not cleaned regularly and that leads to overflow of water from the tank. There complains goes unnoticed. As noted by a woman “We cannot stay, water comes out [from the tank] and it smells bad. And when it rains, it gets mixed with the [drinking] water and then [people] fall sick. We are surviving this way”.

### Food and rations

Women workers, irrespective of the type of contract in private plantations, reported that they were entitled to receive a total of 6 kg ration - 3 kg rice and 3 kg wheat flour after 12 working days. Even children of permanent workers under the age of 16 years were entitled to receive this ration. However, this entitlement was linked with a number of days worked by a worker, and thus prone to the deduction. Almost all women said that they typically receive less than 6 kg (sometimes only 3 kg for 12 working days). They also mentioned that the amount of ration given by plantation estates was not sufficient to feed all the members of the household and therefore they would have to buy additional food from nearby shops.

In addition to this, under the food security programme, the Government entitled each household with a ration card to 5 kg of rice per person. Non-permanent workers receiving ration from government shops noted that shopkeepers would deduct 2-3 kg per household from the entitlement. Apart from this, ration was not distributed regularly. Women also noted that most non- permanent workers in plantations did not have a ration card.

Conucerrently, permanent workers in private plantations reported that they are entitled to firewood from the plantation estates but the quantity of firewood (used as cooking fuel) supplied by plantation estatesonce a year was insufficient. The amount given would last only three months. In government plantations, on the other hand, women reported that distribution of firewood by plantation estate was stopped- for reasons unknown.

#### Intermediary determinants of health

### Health services

Every plantation visited had a hospital; yet women were not satisfied with the health services provided by them. For one, doctors in plantation hospitals were not available round the clock, and workers were mostly referred to the district hospital. In one of the FGDs, the challenge of medicines was raised: “We don’t get all the [prescribed] medicines and we have to buy medicines from outside”. Therefore, for ailments like fever, headache, and body ache, they preferred buying medicines from a nearby private chemist shop.

Permanent workers mentioned that husbands of women workers were not entitled while wives and children of male permanent workers had access to health services in plantations. Therefore, the medical expenditures of husbands (who were not permanent workers) were an additional financial burden.

Non-permanent workers were entitled to health services from plantation hospitals for the period they were engaged in plantations. However, they were not entitled to ambulance services. In contrast, workers in tea plantations covered under Public Private Partnership (PPP), an initiative by the State Health Mission, had access to all health services rendered to permanent workers by the plantation hospital. In light of this, women were happy with the 108 emergency ambulatory service implemented by the State National Health Mission, noting that it has eased transportation of patients to higher health facilities.

All women communicated the feeling that management was not invested in ensuring access to health services. They mentioned that while management was apathetic towards them, politicians were no different. In their experience, politicians showcased their concern towards their community and its well-being only when seeking votes during elections.

*“Whenever a vote is required, XX [name of the political party withheld] party and XX party used to come. Who have given what and who did not give [a vote for them]. [They used to visit] everyone’s house? Now, [forget] at night they don’t even come during daytime, not even once”.*

A similar feeling was expressed regarding doctors and health staff working in plantation hospitals. Recounting several incidents of discrimination, women noted that the doctor simply write the prescription without examining or asking their complaint properly. In fact, in government plantation, women noted that the plantation’s doctor would refuse to touch workers, and prescribe medicines by asking questions from a distance. Further, women across plantations mentioned that they do not have faith in the medicines given by the doctor/health staffin the plantation hospital. For example, a woman noted that “For fever, diarrhea medicine is given and for diarrhea, fever medicine”. They also mentioned that the effect of treatment and medicines prescribed by doctors in plantation and government hospital was slower than the medicines given by a private chemist around the plantation. As mentioned by a non-permanent worker “[We] do not recover quickly [by the medicine prescribed] in a government hospital. Therefore, it is better to spend money on our body and get better quickly”. All women acknowledged financial burden when they or their family representatives were sick.

### Social networks

Women also described a lack of social support in plantations, leading to neglect of their issues by both the family and community at large.. All women reported domestic violence and noted that their husband had beaten them and now it’s a part of their life. While describing the incidents of domestic violence and oppression, women mentioned that they were not able to help each other. A woman in a private tea plantation stated “if we go and ask the husband/family [not to beat the wife], then we will be beaten by our husbands. Who will stand up for us?”

While talking about their onerous life and high daily targets for plucking leaves, women mentioned that eight hours that they spent with their colleagues on the field was the best time of the day. Women narrated how they shared each other’s happiness, sorrow and sang songs while plucking leaves. For instance, a woman at a private plantation said “We don’t remember our worries when we meet each other at work. We laugh, sing and share each other’s pain. We are happy when we are at work”. Women also mentioned helping each other in meeting the day’s target. An unspoken but highly salient bond between women was observed by the researcher at all the tea plantations visited.

#### Individual factors

### Health care use

In plantations, women would delay seeking health care because cost of being sick was substantial: a day’s wage for non-permanent workers and half a day wage for permanent workers. During a discussion in a private plantation, a non –permanent worker had this to say: “whole day she [her colleague] was suffering from severe headache but thinking that everyone will get full payment except her. She came [to work]. No option [for us]”.

Besides this, a few women noted that medicines bought from the chemist (more relied upon than doctors for reasons aforementioned) helped them recover faster and saved them from taking leave.

*“We have to work every day because we will not get money, if we take sick leave for 4-5days. If we do not work continuously for 12 days, then we will not get ration as well. We have to buy everything; we do not have an agricultural field. We are dependent on the plantation. Therefore, we will have to work”.*

*Permanent workers at a government tea plantation.*

### Diet and nutrition

All women noted that they did not have time to think about their diet. Their first concern was to be on time for duty, which required finishing household chores, feeding their children and then making their way to work. Most women either skipped breakfast or – given the pressure to meet daily plucking quotas – skipped lunch, and sometimes both. A few women mentioned that sometimes they skipped their breakfast and lunch as there would not be enough food for everyone in the family: whatever was available, they would cook and keep for their family members, just drink water/tea, and come to work.

In discussions, women mentioned that they knew that for them to be healthy, they should eat nutritious food. They noted that they would like to eat fruits and meat regularly but could not afford either - improving their own nutrition would be at the expense of that of other family members. All women reported eating rice and potatoes regularly and some locally available vegetables.

*“We cannot eat on time. With this wage, can we eat good food by keeping our children hungry? If we think of eating good food, or wear good clothes and [face] cream then it will cost us and we do not have money to spend on these. Tell us, how we will take care of our health?”*

*Permanent workers at a government tea plantation.*

### Alcohol consumption

While alcohol consumption was most prevalent among men, even women in plantations reported drinking. All were aware – in fact acutely – of the harms of drinking. They further noted that it tended to be older women that drank. A woman in the group accepted that she drank alcohol to reduce her body ache after a day’s hard work. Another woman, a single mother, reported that she prepared and sold alcohol from home because her wage was insufficient and this was her additional income.

### Psychosocial stress

We observed a sense of sadness and desperation in the groups while discussing women’s health – as though even having health needs was a luxury. Women narrated that they were living stressful lives with no rest. For all women, a key reason for stress other than family was the fear of losing daily wage. Therefore, the only recourse was to continue working until the body gives up.

## Discussion

This study brings out the spectrum of factors at the individual, intermediate and structural levels that women workers in plantations themselves identified as influencing their physical and mental well-being. Most of these findings, such as poor wages, working and living conditions, health services, education and poverty in plantations are already known. Several studies, media, and even government agencies have documented the poor conditions of workers in tea plantations [7, 8, 19–21], although the emphasis on women worker’s health and well-being has been absent, with a few noteworthy exceptions in other states of India [22, 23].

This study confirms that the causes of women’s health hardships are these social determinants and they have been subject to human and health rights violation. It also adds to the body of evidence in India which shows that the proportion of women in plantation labour has increased [22] but in the context of broader declines in work participation and concomitant decline in working conditions for them, given the steep fall in international tea prices [22, 24]. As Viswanathan and Shah point out in their comprehensive work on women plantation labourers “the crisis as experienced in the plantation sector would have destabilized the livelihoods of the women workforce in particular and their dependent households. Moreover, as plantations are situated in isolated and remote areas, there are no alternative means of supporting their livelihood. Tea planters also increasingly adopted a new strategy of subdividing and fragmenting the plantations into smaller parcels below 10ha so that they could escape from providing the non-wage benefits and the welfare measures as per PLA. This tendency has been on the rise in the plantation sectors, especially in Assam” [24]. Women are therefore trapped in a vicious cycle of ill-health, job insecurity, and penury.

Many of the other findings in this study, like the challenges of eligibility across workers, the lack of financial coverage for health, and the lack of higher education of children was observed in another study with tea plantation workers in Kerala [24]. The irony is that not only are these findings not unique to Assam, many of them have been clearly known for decades, and have been unchanging all the while. Workers are suffering from the same recalcitrant problems, and continue to remain one of the poorest and marginalized populations in the state.

In the plantation sector in Munnar, Kerala, poor working conditions and emoluments were among the root causes for a plantation strike in 2015 by women workers whose demands were ignored by the local trade union [23]. This strike was announced by 10 workers at the male-dominated trade union meeting and followed closely by the media. At its high point, *pempilai orumai* (meaning “female unity” in the native language of the workers, Tamil) had 5000 women protesting the bonus decrease announced by plantation management. The month-long strike had wide ranging impacts – including on tourism in the area- and was successful, even as trade unions carried out retaliatory actions against some of the women. The group is now formalised and has been leveraging for additional concessions and facilities, including emergency ambulatory care [23]. Given that the women in this study also saw each other as a major social support, some of the ingredients for such a mobilisation may perhaps exist in the field site studied.

This is not to suggest that there is no action: in fact, the state government has been seized of and acting on behalf of plantation workers, especially women. The state government has created tea tribe welfare department, and a few existing departments like health, social welfare department, and public health engineering are implementing ongoing national programs in plantation areas. Besides this, the health department has implemented a few dedicated schemes in plantation estates (such as providing financial support for improving health infrastructure in 150 plantation hospitals, and mobile medical units) for improving the health of women [25]. In addition, a few plantation associations such as Assam Branch of Indian Tea Association (ABITA) with support from United Nations International Children’s Emergency Fund (UNICEF) and other Non-Government Organizations have implemented programs in a few plantations for improving maternal, child health and adolescent’s health [26]. However, these approaches to address women’s health in plantations are typically piecemeal, episodic, and do not catalyse the agency of workers themselves, and thus are neither taking a holistic approach to act on social determinants of health (SDH) nor one that can cumulatively do so.

This study revealed that the conditions in which women workers are working and living cannot be ignored if we truly want to improve their health. Evidence suggests that much of the illnesses arise because of the conditions in which people are born and raised [27]. It is also known that at all levels of income, health and illness follow a social gradient – the lower socioeconomic position, the worse the health [27]. Thus, to improve women’s health in plantations, action on the SDH must be taken. But the larger question is who, then, will take the responsibility of improving women’s health in plantation estates? What is the way forward?

### Recommendations

As part of this study, authors engaged in a consultative process of arriving at recommendations for action on SDH.

As is clear from the literature and examples globally, the state government and tea estate sector must transition away from colonial-era policies, mindful of current industry realities and democratic norms. This entails a jurisdictional shift to bring plantation worker’s health, education and other social sectors under the purview of the state through an amendment of the Plantation Labour Act of 1951. Concurrently, the government must enforce PLA provisions requiring tea plantation estates to improve the daily living and working condition of workers.

Second, the government may also choose to claim its moral authority and institute uniformly the mandate of minimum wages for all workers – temporary and permanent, public and private. In addition, unfair deduction if workers are unable to complete the task, or taken entitled leaves or holiday should be removed. Given the primacy given to this determinant by women plantation workers themselves, we feel this may be the most critical determinant to intervene upon, not just in terms of addressing the health of women workers, but also affecting other determinants like access to education and health in the near term.

Third, linked government departments –Education, Tea Tribes, State Rural Livelihood Mission and Assam Skill Development Mission may work together to initiate vocational training of young and old family members of plantation workers and offer higher education scholarships for worker’s children surpassing primary education. Such a move would ensure opportunities for this community to have social mobility beyond plantation estates and also may help widen the economic scope of work and reduce plantation estate dependency. This could also improve and widen the economic base of tea plantation areas and foster growth.

There is a strong need for support of women’s capacity for full participation and representation at workplace and community forums. Self Help Groups have been created in Assam since the late 1990s through a number of centrally funded schemes [28]. These programmes may be extended by departments of women and child development and/or social welfare in plantations with the help of Non-Governmental Organisations. Moreover, tea plantation estates could be enjoined to develop career progression opportunities for women workers while unions could give attention to recruitment of women members and/or having a women’s wing. Alongside this, opportunities for support for women should be brought about, particularly for women affected by violence.

Fifth, in line with the PLA, the tea plantation estates must acknowledge the poor working and living conditions of its workers and invest in providing safe and protective working environment as well as improving housing, water, and sanitation facilities. Improving the living conditions of the non-permanent workers may require land ownership or the opportunity to lease land as due recognition of their labour by plantation estates and government authorities.

It is further suggested that the government must take responsibility for providing health services in plantations, transforming existing plantation hospitals into health and wellness centres – with newly allocated funding under the national Comprehensive Primary Health Care scheme [29]. A key component of the mandate of these centres could be widespread preventive and promotive health services, alongside nutrition, access to alcohol cessation and harm reduction programmes, as well as emphasis on sanitation, hygiene, and occupational safety.

Finally, we reiterate that poverty, poor working conditions,poor social network including unequal gender relation influences health and well-being of an individual and increases inequities in health [30, 31]. Without addressing the structural and intermediary concerns of workers, it may not be enough to intervene to address individual factors such as women workers’ diet and nutrition, accessing health services, alcohol consumption and psychosocial stress. Indeed, there have been critiques in the literature of piecemeal, individual based interventions that do not get at root causes of ill-health [32].

### Limitations

A limitation of this study was the relatively small sample of tea plantations covered. Another limitation is that we did not interview women in age-disaggregated or parity-disaggregated groups. It is possible, likely even, that there may be slight variations in the health needs by these categories. Further, due to resource constraints, our study did not study the range or prevalence of morbidity among health workers using mixed methods, or seek to rank or prioritise SDH beyond their characterisation by workers themselves. Further study may be required to deepen our understanding and guide specific programmatic and policy approaches related to SDH.

## Conclusion

This paper, focusing on structural, intermediary and individual SDH has shown the range of factors that require action to improve the health of women plantation workers in Assam. To have heard from women themselves has been an important step in visibilizing and building accountability for action on the health and social determinants of health of women workers in tea plantation estates in Assam. There is a need to respond to the question posed by these workers that: who will stand up for them? Some very specific actions may be initiated, and larger processes set in motion based on these findings. However, it must be noted that this is just a beginning.

## Data Availability

All FGDs data gathered during this study are included in this published article.

## Abbreviations

FGD: Focus Group Discussion
INR: Indian Rupee
Kg: Kilogram
PLA: Plantation Labour Act
SDH: Social Determinants of Health
USD: United States Dollar
